# Loss of Form

**Published:** 1884-07

**Authors:** 


### LOSS OF FORM.

AFTER women pass middle age they lose a considerable amount of their height, not by stooping, as men do, but by actual collapse, sinking down: this is mainly to be attributed to the perishing of the muscles that support the frame in consequence of habitual and constant pressure of corsets and dependence upon the artificial support by them afforded. Every girl who wears corsets that press upon these muscles and restrict the free development of the fibres that form them, relieving them of their natural duties of supporting the spine, indeed incapacitating them from so doing, may feel sure she is preparing herself to be a dumpy woman. A great pity! Failure of health among women when the vigor of youth passes away is but too patent and but too commonly caused by this practice. Let the man who admires the pieces of pipe that do duty for a human body picture to himself the wasted form and the seamed skin. Most women, from long custom of wearing the corsets, are really unaware how much they are hampered and restricted. A girl of twenty intended by nature to be one of her finest specimens gravely assures one that her corsets are not tight, being exactly the same size as those she was first put into, not perceiving her condemnation in the fact that she has since grown five inches in height and two in shoulder breadth. Her corsets are not too tight because the constant pressure has prevented the natural development of heart and lung space. The dainty waist of the poets is precisely that flexible slimness that is destroyed by corsets. The form resulting from them is not slim, but a piece of pipe, and quite as inflexible.—*Exchange.*

"People, like plants, grow pale and puny if the sun is shut out. Good health is the sunshine of the body; and a cheery disposition is the sunshine of the soul. We build our houses with spacious doors and wide windows, but we shut out the delicious air by closing the doors, and exclude the cheering light and glorious sunshine by inner shutters and two or three layers of curtains."—[DR. W. W. HALL.

